# Building a virtual community of practice: experience from the Canadian foundation for healthcare improvement’s policy circle

**DOI:** 10.1186/s12961-022-00897-0

**Published:** 2022-09-01

**Authors:** Shannon L. Sibbald, Maddison L. Burnet, Bill Callery, Jonathan I. Mitchell

**Affiliations:** 1 School of Health Studies, Faculty of Health Sciences, University of Western Ontario, 1151 Richmond Street, London, ON N6A 3K7 Canada; 2 Department of Family Medicine and the Schulich Interfaculty Program in Public Health, Schulich School of Medicine and Dentistry, London, ON Canada; 3 Healthcare Excellence Canada, Ottawa, ON Canada; 4 Research and Policy, HealthCareCAN, Ottawa, ON Canada

**Keywords:** Virtual communities of practice, Health policy, Value creation, Knowledge exchange, Trust, Networking, Healthcare, Trust

## Abstract

**Background:**

Communities of Practice are formed by people who interact regularly to engage in collective learning in a shared domain of human endeavor. Virtual Communities of Practice (VCoP) are online communities that use the internet to connect people who share a common concern or passion. VCoPs provide a platform to share and enhance knowledge. The Policy Circle is a VCoP that connects mid-career professionals from across Canada who are committed to improving healthcare policy and practice. We wanted to understand the perceived value of the VCoP.

**Methods:**

We used qualitative and quantitative survey research to explore past and current Policy Circle members’ thoughts, feelings, and behaviours related to the program. Our research was guided by the Value Creation Framework proposed by Wenger and colleagues. Three surveys were created in collaboration with stakeholders. Data were analyzed within cohort and in aggregate across cohorts. Qualitative data was analyzed thematically, and quantitative data was analyzed using descriptive statistics (means of ranked and scaled responses).

**Results:**

Survey participation was high among members (Cohort 1: 67%, Cohort 2: 64%). Participants came from a variety of disciplines including medicine, health policy, allied health, and nursing, with most members having a direct role in health services research or practice. The program was successful in helping participants make connections (mean = 2.43 on a scale from 1 to 5: 1 = yes, significantly, 5 = not at all); variances in both qualitative and quantitative data indicated that levels of enthusiasm within the program varied among individuals. Members appreciated the access to resources; quarterly meetings (n = 11/11), and a curated reading list (n = 8/11) were the most valued resources. Participants reported the development of a sense of belonging (mean = 2.29) and facilitated knowledge exchange (mean = 2.43). At the time of this study, participants felt the program had minor impact on their work (mean = 3.5), however a majority of participants (50%) from Cohort 2 planned to acknowledge the program in their professional or academic endeavours. Through reflective responses, participants expressed a desire for continued and deeper professional network development.

**Conclusions:**

The Policy Circle was successful in facilitating knowledge exchange by creating a community that promoted trust, a sense of belonging and a supportive environment. Members were satisfied with the program; to promote further value, the Policy Circle should implement strategies that will continue member participation and networking after the program is finished.

**Supplementary Information:**

The online version contains supplementary material available at 10.1186/s12961-022-00897-0.

## Contributions to the literature

- Virtual Communities of practice (VCoPs) are an increasingly common way to connect individuals with a common interest to share knowledge and network; insufficient evidence exists to support the development and maintenance of VCoPs.
- We introduce facilitators and barriers to successful virtual collaboration between healthcare professionals that can be adopted to increase efficacy of communication for healthcare professionals and the success of VCoPs.
- We offer insight into what mechanisms support the success of a VCoP. Our recommendations will help with the development and support of effective communication online for healthcare professionals. Our findings support an increased understanding and implementation of best practices, current research, and better patient care.
- We identify key indicators for the evaluation of VCoPs that can support future evaluations and cross-program comparisons.

Building a Virtual community of practice: Experience from the Canadian Foundation for Healthcare Improvement’s Policy Circle.

## Background

The concept of Communities of Practice (CoP) was first popularized in the 1990s by Etienne Wenger [1–3]. CoPs are formed by people who interact regularly to engage in collective learning in a shared domain of human endeavor [2]. A community of practice becomes successful over time due to its ability to generate excitement, relevance, and value to attract and engage members [3]. Groups organized as such communities support open dialogue, where members can foster engagement, build common ground, and encourage understanding [4]. Often an overarching aim is to develop a community capable of lasting impact [4]. Wenger supports seven principles for cultivating communities of practice: design for evolution, open dialogue between inside and outside perspectives, invite different levels of participation, develop private and public community spaces, focus on value, combine familiarity and excitement, and create a rhythm for the community [3].

Virtual Communities of Practice (VCoP) are online communities that use the internet to connect people that share a concern or passion and provide a platform where they can share and enhance their knowledge [2]. VCoPs go beyond a website or database, using online learning and engagement methods such as real-time video conferencing to remove geographical barriers and open up the possibility for an interactive and diverse group [5]. VCoPs are used in different disciplines around the world, including social work [6], education [6] and public health [7]. For example, healthcare organizations promote VCoPs as a tool for enhancing knowledge, improving practice, and organizational performance. In this context, it has been found that these communities help alleviate the isolation between healthcare professionals [7]. The promotion of collaborative work in healthcare through VCoPs supports a greater continuum of care for patients and healthcare organizations [7].

Despite the benefits of VCoPs there are some limitations that need to be considered including members fading back or withdrawing from the community [8]. This can be exacerbated by cultural differences hindering communication and community development, the higher possibility of poor and/or superficial online communication, and a lack of response urgency as a result of asynchronous communication [8].

### Building strong VCoPs

Trust and justice have been found to be an important foundation for the success of a VCoP [9–12]. Trust is an antecedent to knowledge sharing in VCoPs across three dimensions: integrity, competency, and benevolence [13]. Issues with trust in VCoPs can significantly impact participation and the development of productive interprofessional relationships in the community [14]. Alternatively, studies show that the importance of the relationship between trust and knowledge sharing decreases over time; as a VCoP matures, other external cues, such as commitment and technology, can enhance knowledge sharing behaviour [11, 15]. Commitment is strengthened when members are inspired, excited, and willing to participate. Active participation is encouraged when issues of trust and privacy are addressed through the creation of an encouraging and open environment [14]. However, there is often a learning curve in obtaining the technological skills needed to participate in virtual communication that may cause barriers and discourage members from joining [14]. For example, an evaluation of the virtual network *InspireNet* in British Columbia reported that nurses and allied health professionals who rated themselves as computer literate still required mentoring and support to adapt to a virtual environment [16].

Leadership plays an important role in ensuring that CoPs and VCoPs develop and achieve a clear vison. VCoP leaders ultimately influence the structure and help shape a common vision [14, 17]. This is especially true during the VCoP launching stage when leadership should consider environment, objectives and the appropriate size of the community [18, 19]. Optimal size of the community should align with the aims of the VCoP and methods that work best for knowledge sharing [20]. In the context of collaborative development, as group size increases productivity decreases; a group size of seven has been shown to be optimal [21].

Two commonly cited models to support the development or maintenance of VCoPs in the healthcare sector are the Integrated VCoPs Success Model [22] and the integrated conceptual model [23], proposed by Alali and Salim in 2011 and 2013, respectively. The integrated conceptual model says that a VCoP’s knowledge quality, system quality, service quality, perceived usefulness, and perceived ease of use all positively affect a member’s satisfaction within the VCoP. Members’ satisfaction in turn positively affects their knowledge sharing behaviour and improves the overall effectiveness of the VCoP [23]. The Integrated VCoPs Success Model provides a solid theoretical background to Alali and Salim’s integrated conceptual model [22]. It includes socio-psychological factors (such as attitude, motivations, and trust), and technological factors (such as perceived ease of use and knowledge content quality) as main indicators to measure the success of VCoPs [22].

VCoPs are very similar to online learning environments. In his work on web-based learning environments, Renaud [24] discusses three elements that are important for learning to occur: time, space, and tools. Renaud [24] further outlined seven steps to building a successful online learning environment: (1) clearly identify the purpose, (2) create a distinctive gathering place, (3) promote effective leadership from within, (4) define norms and a code of conduct, (5) allow for a range of member roles, (6) allow for and facilitate subgroups, and (7) allow members to resolve their own disputes. Applied to VCoP, these concepts support the development and maintenance of successful VCoPs.

### Evaluating VCoPs

Evaluations of VCoPs should be done using a range of indicators and a combination of qualitative and quantitative methods [25]. Frameworks can help ground the evaluation process to provide legitimacy, relevance, and cohesion to evaluation claims [26]. Various frameworks suggest different measures (e.g., personal reflections, questionnaires) and indicators (e.g., trust, tech challenges) to evaluate VCoPs [25–27].

The Value Creation Framework developed by Wenger et al. [25] is an evaluation framework that assesses the value of learning or extent of knowledge-sharing that is enabled by communities. It focuses on the dynamic relationship of five values that are produced when people come together for knowledge sharing [25]: (1) immediate value (focuses on the experiences, activities, and interactions that members have in the community); (2) potential value (focuses on what was produced by the VCoP, such as knowledge capital); (3) Applied value (examines changes in practice); (4) Realized value (refers to organizational and individual performance); (5) Reframing value (looks at the VCoPs definition of success) [25]. This framework has been used in the evaluation of VCoPs [26–29]. For example, Cowan and Menchaca [27] conducted an analysis of the internet-based Master in Educational Technology (iMET) program at Sacramento University using the Value Creation Framework. They outlined how the value creation within the iMET program had supported the sustainability of this successful VCoP over 13 years [27]. We chose to use this framework in our analysis based on its nuanced assessment of value creation from knowledge sharing, and successful application across a range of similar VCoPs. Successful VCoPs do not need to achieve each value in the Value Creation Framework; different stakeholders are often interested in different interpretations of success and values [25]. The Evaluation Framework for Extra-Organizational Communities of Practice mitigates this limitation by providing a comprehensive multi-level tool for categorizing the values generated in VCoPs and determining if the absence or presence of a value affects outcomes [30].

The main objective of most VCoPs is to facilitate knowledge sharing between members. However, achieving a continuous exchange of knowledge is a challenge of VCoPs in health [7]. In this study we wanted to explore the facilitators and barriers for continuous knowledge exchange within a newly formed VCoP as well as determine the level of the program’s impact over the past two years. Our aim was to provide recommendations to improve the program for future cohorts.

### Case study—A VCoP in action: the policy circle

We set out to evaluate a VCoP and to explore the facilitators and barriers to its success. The Policy Circle, a part of the former Canadian Foundation for Healthcare Improvement (CFHI), now Healthcare Excellence Canada (HEC), connects mid-career professionals from across Canada who are committed to improving healthcare policy and practice [31]. The central aim of the CFHI Policy Circle was to foster and contribute to the growth of the next generation of healthcare leaders. This competitive year-long program provides opportunities to take advantage of CFHI programming, to participate in virtual mentoring, and to collaborate with peers. Through the Policy Circle, members gain access to a curated resource listing (academic research, literature, and newsletters), the Institute for Healthcare Improvement’s (IHI) online courses, and receive financial support to attend a national event or conference [31]. One of the most important aspects of the Policy Circle is the regular opportunities to connect with and learn from peers and healthcare leaders across Canada [31]. Quarterly meetings bring Policy Circle members together to share stories and learn from each other’s experiences, as well as discuss “hot topics” and current events. Each Policy Circle member is matched with a mentor who is aligned with their interests or goals and has extensive policy and practice experience and expertise. Members have access to additional opportunities that arise organically throughout the year.

## Objectives

As the program concluded its second year in 2021, we wanted to explore what, if any, value the CFHI Policy Circle created as a social learning activity. Our main objective in reviewing the Policy Circle were to identifying barriers and facilitators, to determine the level of the program’s impact over the past two years, and to improve the program for future cohorts. In order to achieve our objectives, we sought to determine whether each of the values introduced by Wenger and colleagues [25] were created during the Policy Circle’s life course by answering value-specific research questions. A table of Wenger et al.’s [25] value domains, associated research question, and indicators are listed in Table 1. Based on our findings we suggest program improvements that can be implemented for future cohorts.

**Table 1:** Value creation framework: research questions and indicators

Value created	Research question	Indicators
Immediate value	Are the activities of the program perceived to have value in itself?	Levels of participation Levels of engagement Value of participation Networking
Potential value	Was there an increase in knowledge capital through the promotion of knowledge exchange?	Level of trust Reputation of the community Skills acquired Information received
Applied value	Did the program facilitate changes in practice?	Implementation of advice, outputs, and insights by members into their practice
Realized value	Did the program perform up to the expectations of members?	Personal performance Organizational performance
Reframing value	Following program completion, did the definition of success change for members?	Relationship with stakeholders

## Methods

We used a mix of qualitative and quantitative survey research, guided by the Value Creation Framework [25], to explore past and current Policy Circle members’ thoughts, feelings, and behaviours. We used Qualtrics, an online survey software tool to support the design, collection, and analysis of the surveys [32]. Three surveys with Likert scales and open-ended options were created to capture the experiences of two cohorts that participated in the program.

### Participants and sample

The first cohort of the CFHI Policy Circle (PC-C1) ran its inaugural year with six participants from April 2019 to March 2020. This cohort was surveyed retrospectively in February 2021. The second cohort (PC-C2) (April 2020–March 2021) had 11 members that were asked to complete a similar survey at two time points: mid-way and at program completion. For both cohorts, Policy Circle members applied to the program through an online application. Each application was reviewed by a pair of reviewers drawn randomly from a pool of four internal reviewers and two external reviewers. Based on reviewer results and recommendations, CFHI’s CEO made the final selection of Policy Circle members. Participants came from a variety of disciplines including medicine, health policy, allied health, and nursing. Participants' primary work was varied, with most members having a direct role in health services practice or research. Demographics for Policy Circle members from both cohorts are presented in Appendix 1.

### Survey development

Survey development was collaborative, and based on the Value Creation Framework [25]; questions were designed with the indicators of each value in mind and discussed with the evaluation group (SLS, BC, and JM). Face and content validity were met through the iterative co-development process of the surveys. Open ended questions were included to explore organic reflections that may not align with the framework, as well to determine if the program had achieved the member’s expectations.

In addition, the PC-C2 survey included aggregate and ranking responses based on the midpoint evaluation findings. This allowed us to explore shared goals as well shifting perspectives over time (see Additional file 1 for all three surveys).

### Survey distribution

All surveys were sent out to members’ emails using an anonymous link automated by the Qualtrics software. Participation in the survey was voluntary and done as a collaborative quality improvement effort, therefore consent was not explicitly obtained. A disclosure statement was placed at the beginning of each survey and members were provided with the email addresses of SS and BC in case of any questions. PC-C1 was sent the retrospective survey in February 2021 and a reminder email was sent out after two weeks. The midpoint survey (PC-C2-M) for PC-C2 was sent out in November 2020 and the exit survey (PC-C2-E) was sent to members after the last quarterly meeting (March 2021), each with a reminder email sent out after two weeks.

### Data analysis

Data was analysed in within cohort and in aggregate across cohorts using indicators that represented each of the values presented by Wenger and colleagues [25]. Qualitative data was analyzed through thematic analysis (identifying common themes in member responses) by two members of the project team (MB, SS). Preliminary results were discussed with the whole project team and presented back to the Policy Circle Cohort 2; discrepancies were resolved through discussion and feedback. We used descriptive statistics in the analysis of quantitative data. Survey questions answered on a scale were interpreted using the mean score and the standard deviation of the responses. Ranked data was analysed using the mean ranking for each category, where the answer with the lowest mean was ranked as 1.

## Results

Four out of six members from Cohort 1 responded to the retrospective survey (67% response rate). From Cohort 2, nine out of 11 members provided responses for the midpoint survey (81.8% response rate) and seven responded to the exit survey (63.6% response rate).

### Activities and interactions

Looking at both cohorts, all members indicated that they had attended quarterly meetings; 73% of all respondents (*n* = 8/11) attended three or more times (PC-C1: 2/4 (50%), PC-C2-E: 6/7 (86%)) and the remainder attended one to two times (*n* = 3/11; 27%). Six members (55%) participated in the mentorship meetings (PC-C1: 2/4 (50%), PC-C2-E: 4/7 (57%)). Making new connections was a key goal of the program with a mean score of 2.43 (SD = 1.5) (PC-C2).

We examined members’ perceived value of participation and connections within the program. When asked if members enjoyed the program and opportunities on a scale 1 to 5, the average mean score was 2.71 (SD = 1.39), with two out of seven members answering 1 (yes, significantly). In personal reflections from both cohorts, member statements included, “*I’m honoured to be affiliated with a diverse, highly motivated and ambitious group of future leaders in Canadian healthcare policy*”, “*I really loved being a part of this group”,* and “*I found the areas I did participate extremely useful.”.*

In comparing the number of individuals from Cohort 2 who took advantage of the program’s opportunities at midpoint versus at exit, there was no change aside from an increase for IHI open school (Table 2). On exit, five of seven members (71%) reflected on how their engagement and enthusiasm changed throughout the program. Three of the five individuals (60%) reported it had not changed, one stating “*my expectations were fairly high entering the program; I believe those expectations were met.*” One individual said it declined, while another reported an increase stating, “*the groups enthusiasm was contagious.”*

**Table 2:** Use of opportunities: Cohort 2- Midpoint vs. Exit

	PC-C2-Midpoint (*n* = 9)			PC-C2-Exit (*n* = 7)	
	Yes	No	Not Yet	Yes	No
Curated resource listing	77.8% (*n* = 7)	11.1% (*n* = 1)	11.1% (*n* = 1)	71.4% (*n* = 5)	28.6% (*n* = 2)
Mentorship meetings	66.7% (*n* = 6)	11.1% (*n* = 1)	22.2% (*n* = 2)	57.1% (*n* = 4)	42.9% (*n* = 3)
Quarterly meetings	100% (*n* = 9)			100% (*n* = 7)	
IHI open school	33.3% (*n* = 3)	22.2% (*n* = 2)	44.4% (*n* = 4)	71.4% (*n* = 5)	28.6% (*n* = 2)

Data was taken from Cohort 2's midpoint and exit surveys. Percentages (%) were rounded to the nearest tenth

### Knowledge capital

The Policy Circle program gave members access to numerous opportunities and resources (Tables 1, 2). Following the quarterly meetings, the curated resource listing was the most used resource by members (PC-C1: 3/4 (75%), PC-C2: 5/7 (71%)), used on average two times per person. In the context of Cohort 2, three of the nine members that completed the midpoint survey 3 (33%) participated in the IHI open school; this increased to 5 members the exit survey (Table 3). The journal club, the CPSI/CFHI Branding exercise and, volunteer judging for the CFHI innovation challenges were three opportunities only available to Cohort 2. At the midpoint 66% of respondents (*n* = 6/9) participated in the journal club and 33% (*n* = 3/9) volunteered to participate in an additional opportunity offered by the CFHI. On exit, all seven respondents participated in the journal club and four participated in an additional opportunity. Only two members from either cohort (one from each cohort) attended a conference.

**Table 3:** Use of opportunities: cohort 1 vs cohort 2

	PC-C1 (*n* = 4)			PC-C2-Exit (n = 7)		
	0	1–2	3 +	0	1–2	3 +
Curated resource listing	25% (*n* = 1)	50% (*n* = 2)	25% (*n* = 1)	28.6% (*n* = 2)	42.9% (*n* = 3)	28.6% (*n* = 2)
Mentorship meetings	50% (*n* = 2)	25% (*n* = 1)	25% (*n* = 1)	42.9% (*n* = 3)	42.9% (*n* = 3)	14.3% (*n* = 1)
Quarterly meetings		50% (*n* = 2)	50% (*n* = 2)		14.3% (*n* = 1)	85.7% (*n* = 6)
IHI open school	75% (*n* = 3)	25% (*n* = 1)		28.6% (*n* = 2)	57.1% (*n* = 4)	14.3% (*n* = 1)

Data was taken from PC-C1 and PC-C2-Exit to keep comparison consistent. Percentages were rounded to nearest tenth

When measuring potential value we asked members in PC-C2-E if the program was successful in facilitating increased knowledge and skills, on a scale from 1 (yes, significantly) to 5 (no, not at all). All members either answered 1, 2 or 3 with a mean score of 2.43 (SD = 0.73). Further, “ability to advance knowledge” was reported as the third most important strength of the program.

All members answered either in the affirmative or neutral when asked if they are proud to tell others they are a Policy Circle member (100% of PC-C2 answered yes and 50% from PC-C1 answered yes) (rated on a 5-point likert from 1 = Yes, 5 = No). Members also expressed trust in their colleagues' abilities, stating, “*I liked being connected to like-minded professionals in a similar stage of career outside my institution*”, “*those connections and conversations at meetings were professionally and personally rewarding*” and “*the policy circle members were great in providing advice.*” We asked participants in PC-C2-E to rate whether the program helped them achieve a sense of belonging along the same scale. The mean rating was 2.29 (SD = 1.03). Some members also added descriptively, “*I felt this group provided a safe space to share ‘work challenges’*” and that the Policy Circle program “*created a sense of community when we were all relatively isolated*” (referring to the challenges of COVID-19).

### Changes in practice

We asked PC-C1 and PC-C2-E if their work was positively impacted by their participation in the Policy Circle (ratings were from 1 = Yes, significantly, 3 = Yes, somewhat, 5 = No, not at all), nearly half said at least somewhat with the mean response of 3.5 (SD = 1.35) and one of 11 members (9%) answered “yes, significantly.” In open ended responses participants described ways in which work was impacted including connecting with a “*new group of researchers”* and helping form “*a more national network of people to assist with my work.*” Five respondents described some barriers to realizing an impact including, “*no opportunities have arisen”, “I haven’t been able to connect with my assigned mentor”,* and that many external colleagues are unaware of the program which “*made it hard to share.”*

Half (*n* = 2) of respondents from PC-C1 said they acknowledge their Policy Circle experience in their current work through informal means such as including it in their *LinkedIn* profile. Nearly all respondents to PC-C2-E (*n* = 6; 86%) planned to acknowledge the program in their current or future work by listing it on their CV, promoting and referring the program to others, and continuing to connect with the CFHI (now HEC).

### Organizational and individual performance improvement

Several key strengths of the program were identified, including collaboration with colleagues and the CFHI, formal mentor relationships, knowledge sharing, good organizational structure, and accessing different perspectives. When asked to rate the relative importance of the strengths of the program (done from most to least important (1 = most, 5 = least), networking was rated as the most important strength by members, followed by the program coordinator for the Policy Circle (mean = 2.43, SD = 0.90), ability to advance knowledge (mean = 2.57, SD = 1.05) and mentorship (mean = 3.14, SD = 1.25).

PC-C2-M asked members openly what could be done to improve the program. For PC-C2-E, we summarized answers and had members rank them from most to least important (ranked improvement suggestions from most to least important (1 = most important, 8 = least important). Adding face-to-face meetings was most commonly ranked first (mean = 3.57, SD = 2.82) and inviting a guest speaker that members would not get regular access to was rated most important (mean = 3, SD = 1.85). Secondly, having a formal contribution to policy that members can work on together throughout the year, with a mean of 3.14 (SD = 1.73), followed by extra optional meetings that focus on a specific topic (mean = 3.43, SD = 2.26). Other improvement suggestions in order of ranking are having a collective goal from the start (mean = 3.71, SD = 1.39), more structure and linkages to different Canadian sector leaders in order to facilitate cross context engagement (mean = 4.14, SD = 1.36), and providing more information on how to access opportunities (mean = 4.86, SD = 2.03).

### Redefining

We asked members for ideas on continuing support as a Policy Circle alumni. Participants most commonly cited having opportunities to continue to engage with the CFHI (now HEC) and current, past, and future Policy Circle members to facilitate networking and collaboration. Members suggested annual meetings or get togethers that include an overlap between cohorts. Other suggestions included setting clear objectives for the gathering at future meetings and having continued access to resources like the IHI open school.

## Discussion

### Immediate value: activities and interactions

Participation in the activities of the program (e.g., quarterly meetings and associated opportunities) were high. This finding can possibly be attributed to the use of familiar and reliable technology by the Policy Circle (the program utilized *Microsoft Teams*). According to McLoughlin et al. [14] utilizing synchronous communication through familiar technology builds greater confidence and in turn increases participation and commitment to the VCoP. All members of PC-C2 hoped to continue their engagement with the program, suggesting that members are strongly committed to the program, and are inspired, excited and willing to continue their participation [14]. High levels of commitment can explain the increasing trend reported in participation of the associated opportunities (IHI open school), between PC-C2-M and PC-C2-E. This finding is consistent with that of Chang et al. [11], who stated that the role of commitment on knowledge sharing behaviour becomes more important over the life course of a VCoP [11]. Qualitative responses from participants strongly suggested that members enjoyed the program, while the quantitative data showed a greater variance between answers. This discrepancy may be due to individual expectations that impact levels of enthusiasm and engagement. While there was an increasing trend in participation, there was no change in the level of engagement between PC-C2-M and PC-C2-E. C1 and C2 had the same level of engagement compared to each other. This suggests that level of engagement does not correlate to level of participation, and further supports the importance of the relationship between commitment and knowledge sharing as VCoPs mature [11]. As well, targeted reminders from the program coordinator to individual members to increase uptake of Policy Circle activities may have helped increase engagement.

High levels of participation (and VCoP success) can also be attributed to the successful networking that occurred within the program. According to literature, networking has been found to be motivator for participation in knowledge sharing as it can lead to career advancement and enhancement of professional reputation [33–36]. Overall, the program’s activities were perceived to have value by participants.

### Potential value: knowledge capital

Members took advantage of the opportunities offered by the Policy Circle program, suggesting that some level of information and skills were acquired by members. A high percentage of Cohort 2 participants attending journal club (*n* = 7, 100%) by the time they exited the program indicated participants wanted more frequent meetings outside of current quarterly meetings.

Overall, the VCoP was successful in achieving its main objective. When members were asked if that program was successful in facilitating increased knowledge and skills the mean score was in the affirmative, while ability to exchange knowledge was rated in the top three strengths of the program. This indicates that a positive level of knowledge exchange and skill development had occurred.

The majority of members were proud to tell others that they were a part of the Policy Circle program. This suggests that members believed the program’s reputation is credible and that the values of the community aligned with their own. This aligns with the concept of integrity-based trust [13]. Further members also expressed trust in their colleagues’ abilities, suggesting the presence of competence-based trust [13]. Usoro et al. [13] found that integrity, competence, and benevolence-based trust are positively related to knowledge sharing behaviour. Trust also plays a multidimensional role in enabling the free flow of creativity by creating a socially supportive environment that supports knowledge sharing [10]. We found that members believed the program had a supportive environment that maintained a positive sense of community and belonging. This aligns with the concept of benevolence trust and suggests the program facilitated a psychological safe space.

### Applied value: changes in practice

When asked whether their work was positively impacted by their participation in the Policy Circle, the majority of members described barriers to impact. One study found that if individuals do not find value in the community, levels of engagement and participation decrease [37]. This possibly explains the observed differences between PC-C1 and PC-C2-E when asked about acknowledgement of the Policy Circle. Whereas the majority from Cohort 2 planned to acknowledge the program in their work, only half from Cohort 1 currently do so. As time passes without the opportunity to continue engagement, participation and acknowledgement may decrease. Overall, the program has had minimal perceived impact on practice.

### Realized value: organizational and individual performance improvement

The overall consensus was that members were highly satisfied with their participation in the program based on the reported strengths of the program. Our results are consistent with Alali and Salim’s integrated conceptual model [23] and we can assume that the reported strengths facilitated knowledge exchange within The Policy Circle. For example, aspiring to work together as a harmonious team has been found as a common theme in VCoPs, and adequate leadership seems to be a factor to this success [38]. In the Policy Circle VCoP the program coordinator was a top ranked strength and in turn promoted member satisfaction and overall success [22]. The leadership in the program further empowered members to lead various initiatives (such as the Journal Club) which contributed to the growth and commitment to the program.

A common theme from participant suggestions for program improvement was a focus on connection between members and other external stakeholders beyond the duration of the program. This suggests that members wanted opportunities to make deeper connections. This limitation may be attributed to the impact of COVID-19, as the events of the pandemic restricted the world from face-to-face interaction. Mcloughlin et al. [14] pointed out that the development of relationships in VCoPs are critical to the development of trust through in-person interactions; thus, the inability of Policy Circle members to have met in-person may be why participants felt that deeper connections could have been made. However, a recent study found that connecting virtually is a suitable alternative that can help mitigate the negative effects of the COVID-19 pandemic [39].

### Reframing value: redefining

Participants in both cohorts reported a sense of belonging and community during the year long program; however, Cohort 1 reported feeling disconnected from the Policy Circle program and the CFHI after their year-long program ended. This observation is consistent with Shaheen et al.’s findings that some participants from their VCoP felt forgotten despite reporting a sense of belonging [39]. This suggests that organizations running VCoPs, including the CFHI (now HEC), should consider support for alumni as a way to redefine success following program completion.

### Limitations

The surveys allowed for responses that were directly reflective of participant thoughts, feelings, and behaviours. The small sample may be considered a limitation in the current context, however this was mitigated to the extent possible by the high response rates for both cohort groups to all three questionnaires. We presented our data in aggregate form and compared responses across cohorts—this was a strength as it allowed us to gain a better understanding of the results. Some questions and responses were cohort specific, therefore we cannot generalize all answers across cohorts. However, in the context of quality improvement, this study still provided significant findings for the program. Secondly, the lack of pilot testing of our surveys may be considered a limitation in the context of validity. Since our survey was developed in collaboration with key stakeholders (including members of the Policy Circle), we believe this is limitation was mitigated. Due to the small sample size, we did not consider the role of the participant’s demographics in their responses. This may be considered a limitation as age or gender may impact how the participant responded, however the small sample size did not allow for any statistically significant analyses based on disaggregated demographic data. In addition, since the Policy Circle application process does not consider age or gender, this analysis would not significantly affect the results in terms of quality improvement. The time frame of this study occurred during the COVID-19 pandemic, however since Cohort 1 ran before the pandemic the impact of the pandemic was not considered in all aspects of our analysis. Finally, while we cannot state the direction of the relationship between facilitating variables and success, we can state that a relationship exists, and it should be considered in VCoP success.

## Conclusions

Our evaluation of the Policy Circle determined that the program was successful in facilitating knowledge exchange and network development among its members. This is partially due to the presence of trust, a sense of belonging, and a supportive environment within the program. Participants felt that there was the possibility to create deeper connections with other members and the organization during and after the program. As the Policy Circle continues to mature, stakeholders should implement strategies that facilitate VCoP impact on the practice. Strategies to keep members involved beyond the one year should also be considered; especially initiatives that foster leadership by current members. We recommend that the program facilitates cross-cohort collaboration with past and future participants, as well as health policy leaders. Our recommendations our consistent with Li et al.’s suggestion to focus on specific characteristics, such as support for member interaction, knowledge sharing and building a sense of a belonging, that facilitate interventions that support relationship building to optimize the function of these groups [39].

Our findings demonstrate the value of the different components of the Policy Circle program, and describe factors that have contributed to its success. The CFHI (now HEC) can use these findings in the development of future Policy Circle programs to help achieve HEC’s goals of fostering the growth of future healthcare leaders and a greater impact on improvements in healthcare. Further, during the creation of similar programs, our recommendations can help stakeholders identify which factors should be implemented and what possible barriers there may be. We provide a real-life example that supports the current literature on VCoPs and adds to existing evidence of evaluations of VCoPs. We identify appropriate indicators based on a recognized framework that measure the value produced; these indicators can support future evaluations as well as cross program comparisons. This study highlights the current limitations known about VCoPs as well; moving forward future research on the appropriate duration of VCoP Cohorts and the impact of member demographics (e.g., gender and age) on a VCoP should be conducted to ensure that optimal outcomes are being achieved. As well, research on methods of communication and the difference in achieving successful outcomes between virtual and in person communities should be conducted.

### Supplementary Information

**Additional file 1. **Data reports.

**Additional file 2. **Reporting standards checklist.

## Data Availability

All data generated and analyzed during this study are included in this published article as Additional files 1, 2.

## Appendix 1: The Policy Circle Demographic Chart for all members in Cohorts 1 and 2

**Table:**

Demographic	Members (n) (%)
*Gender*	
Male	4 (24%)
Female	13 (76%)
*Geographies*	
Western Canada	3 (18%)
Central Canada	12 (71%)
Northern Territories of Canada	1 (6%)
United States	1 (6%)
*Degrees Acquired**	
MHSc	2 (12%)
MSc	3 (18%)
MA	4 (24%)
MBA	3 (18%)
PHD	8 (47%)
MD	2 (12%)
Unlisted	2 (12%)
*Discipline**	
Nursing	1 (6%)
Medicine	1 (6%)
Allied Health	7 (41%)
Health Policy	7 (41%)
Health Research	7 (41%)

*Members fell into more than one category

## Abbreviations

CoP: Communities of Practice
VCoP: Virtual Communities of Practice
CFHI: Canadian Foundation for Healthcare Improvement
HEC: Healthcare Excellence Canada
IHI: Institute for Healthcare Improvement
PC-C1: Policy Circle-Cohort 1
PC-C2: Policy Circle-Cohort 2
PC-C2-M: Policy Circle-Cohort 2-Midpoint Survey
PC-C2-E: Policy Circle-Cohort 2-Exit Survey

## References

[1] Wenger E (1998). Communities of practice: Learning, meaning, and identity.

[2] Wenger E, Wenger-Trayner B. Communities of practice a brief introduction. 2015. https://wenger-trayner.com/wp-content/uploads/2015/04/07-Brief-introduction-to-communities-of-practice.pdf

[3] Wenger E, McDermott R, Snyder WM (2002). Cultivating Communities of Practice: A Guide to Managing Knowledge.

[4] Hayes C. Policy Circles: Their Influence and Possibilities – Mackinac Center. 2019. https://www.mackinac.org/policy-circles-their-influence-and-possibilities. Accessed 26 July 2020.

[5] Bermejo-Caja C, Koatz D, Orrego C (2019). Acceptability and feasibility of a virtual community of practice to primary care professionals regarding patient empowerment: a qualitative pilot study. BMC Health Serv Res.

[6] Cook-Craig PG, Sabah Y (2009). The role of virtual communities of practice in supporting collaborative learning among social workers. Br J Soc Work.

[7] Bond MA, Lockee BB (2014). Building Virtual Communities of Practice for Distance Educators.

[8] Saigi-Rubio F, Gonzalez-Gonzelez I. Cooperative Learning Environments: Virtual Communities of Practice in the Healthcare Sector. eLC Research Paper Series. 2014; 9, 15–26. https://raco.cat/index.php/eLearn/article/view/305375/395208

[9] Johnson CM (2001). A survey of current research on online communities of practice. Internet Higher Educ.

[10] Fang YH, Chiu CM (2010). In justice we trust: Exploring knowledge-sharing continuance intentions in virtual communities of practice. Comput Hum Behav.

[11] Askay DA, Spivack AJ (2010). The multidimensional role of trust in enabling creativity within virtual communities of practice: A theoretical model integrating swift, knowledge-based, institution-based, and organizational trust. Proc Ann Hawaii Int Conf Syst Sci.

[12] Chang CM, Hsu MH, Lee YJ (2015). Factors influencing knowledge-sharing behavior in virtual communities: a longitudinal investigation. Inf Syst Manag.

[13] Hernandes CA, Fresneda PS. Main Critical Success Factors for the Establishment and Operation of Virtual Communities of Practice. 2003. http://citeseerx.ist.psu.edu/viewdoc/download?doi=10.1.1.197.2248&rep=rep1&type=pdf

[14] Usoro A, Sharratt MW, Tsui E, Shekhar S (2006). Trust as an antecedent to knowledge sharing in virtual communities of practice. Knowl Manag Res Pract.

[15] Mcloughlin C, Patel KD, O'Callaghan T, Reeves S (2017). The use of virtual communities of practice to improve interprofessional colloboration and education: findings from an integrated review. J Interprof Care.

[16] Trees L. KM Communities are Classic, and Classic Never Fades Away. 2020. https://www.apqc.org/blog/km-communities-are-classic-and-classics-never-fade-away?mkt_tok=eyJpIjoiWm1aak9UYzJPRGhpWmpGbSIsInQiOiJFUVVMV2dRMFlRZDNVd1wvV0RLd0Q1U0djM2J4V21jNVpzTUZybWhBUU5XZ0liR0hjcllhaDd2S1p5RFdsTnRub2VrRSt6UnJ3bnhaWTZ5TEYydW4yeUJjZjRhN0tNSjkyZDhCTXBNWG51NENsSXc5K1FvV3FQbGdQZkZ6amRjMGEifQ%3D%3D

[17] Frisch N, Atherton P, Borycki E, Mickelson G, Cordeiro J, Lauscher HN, Black A (2014). Growing a professional network to over 3000 members in less than 4 years: evaluation of InspireNet, British Columbia's virtual nursing health services research network. J Med Internet Res.

[18] Bourhis A, Dubé L, Jacob R (2005). The Success of Virtual Communities of Practice: The Leadership Factor. Electron J Knowl Manag.

[19] Ardichvili A (2008). Learning and knowledge sharing in virtual communities of practice: motivators, barriers, and enablers. Adv Dev Hum Resour.

[20] Dubé L, Bourhis A, Jacob R (2005). The impact of structuring characteristics on the launching of virtual communities of practice. J Organ Chang Manag.

[21] Li LC, Grimshaw JM, Nielsen C (2009). Evolution of Wenger's concept of community of practice. Implement Sci.

[22] Renger M, Kolfschoten GL, de Vreede GJ (2008). Challenges in collaborative modelling: a literature review and research agenda. Int J Simul Process Model.

[23] Alali H, Salim J. Information system success and acceptance theories: Towards developing a “virtual communities of practice” success model. In: 2011 International Conference on Semantic Technology and Information Retrieval, STAIR 2011. 2011; doi: 10.1109/STAIR.2011.5995807

[24] Alali H, Salim J (2013). Virtual communities of practice success model to support knowledge sharing behaviour in healthcare sector. Procedia Technol.

[25] Renaud J. Online Orientation and Team Building. 2020.

[26] Wenger E, Trayner B, de Laat M. Promoting and assessing value creation in communities and networks: a conceptual framework. Rapport 18, Ruud de Moor Centrum, Open University of the Netherlands; 2011

[27] Catherine. Evaluating Communities of Practice & Social Learning. 2017. https://actionevaluation.org/thoughts-evaluating-communities-practice-social-learning/ Accessed 27 Oct 2020

[28] Cowan JE, Menchaca MP (2014). Investigating value creation in a community of practice with social network analysis in a hybrid online graduate education program. Distance Educ.

[29] Booth SS, Kellogg SB (2015). Value creation in online communities for educators. Br J Edu Technol.

[30] Mckellar KA, Berta W, Cockerill R, Cole DC, Saint-Charles J (2020). (Krystal)Application of an Evaluation Framework for Extra-Organizational Communities of Practice: Assessment and Refinement. Can J Prog Eval.

[31] Canadian Foundation for Healthcare Improvement (CFHI). https://www.cfhi-fcass.ca/what-we-do/shape-a-better-future-for-healthcare/policy-circle. Accessed 26 July 2020.

[32] Qualtrics. Online survey software - Powering +1B SURVEYS Annually. 2020. https://www.qualtrics.com/core-xm/survey-software/. Accessed 19 Feb 2021.

[33] Ardichvili A, Page V, Wentling T (2003). Motivation and barriers to participation in virtual knowledge sharing teams. J Knowl Manag.

[34] Ardichvili A, Maurer M, Li W, Wentling T, Stuedemann R (2006). Cultural influences on knowledge sharing through online communities of practice. J Knowl Manag.

[35] McLure-Wasko M, Faraj S (2005). Why should I share? Examining social capital and knowledge contribution in electronic networks of practice. MIS Q.

[36] Scarbrough H (2003). Why your employees don’t share what they know. KM Review.

[37] Haas A, Abonneau D, Borzillo S, Guillaume LP (2020). Afraid of engagement?. Towards an understanding of engagement in virtual communities of practice.

[38] Shaheen Q, Kothari A, Conklin J, Sibbald S (2021). Supporting successful communities of practice for older adults: a qualitative secondary analysis. Educ Gerontol.

[39] Li LC, Grimshaw JM, Nielsen C (2009). Evolution of Wenger's concept of community of practice. Implementation Sci.

[40] Ogrinc G, Davies L, Goodman D, Batalden P, Davidoff F, Stevens D. SQUIRE 2.0 (Standards for Quality Improvement Reporting Excellence): revised publication guidelines from a detailed consensus process. 2015 10.4037/ajcc2015455 26523003

