# Supplementary material for: Building a virtual community of practice: experience from the Canadian foundation for healthcare improvement’s policy circle
Source: Health Res Policy Syst. 2022 Sep 1;20:95. doi: 10.1186/s12961-022-00897-0 (PMC9434556; doi:10.1186/s12961-022-00897-0)
Supplement: Supplementary file 1 — Additional file 1. Data reports. [file 12961_2022_897_MOESM1_ESM.pdf]

PC-C1 - Summary

Start of Block: Default Question Block

Q0
Thank you for taking the time to complete this survey. We are collecting this information as a quality improvement and self-evaluation exercise. You do not have to complete any questions you do not want to. There is no direct benefit to completing the survey, other than the chance to be reflective of your previous (and current) engagement with the Policy Circle. Your individual results will not be shared. All data will be collected and reported in aggregate. .
If you do have any questions, please reach out to Shannon (ssibbald@uwo.ca) or Bill (bill.callery@cfhi-fcass.ca).

Q1 Please explain what the Policy Circle is from your perspective.

____

____

____

____

____

Q2 In order, please list your top three goals for participating in the policy circle?

○ 1. (1) ____

○ 2. (2) ____

○ 3. (3) ____

Q3 How many times (approx.) did you take advantage of the following Policy Circle opportunities?

	Number of times		
	0 (1)	1-2 (2)	3+ (3)
Harkness readings (1)	○	○	○
Mentorship meetings (2)	○	○	○
Quarterly meetings (3)	○	○	○
IHI Open school (5)	○	○	○
Other (6)	○	○	○

Q4 If you selected other - please explain:

____

____

____

____

____

Q5 Did you attend a conference?

○ Yes (1)

○ No (2)

Q6 If yes - which conference did you attend?

____

Q7 How have you continued your engagement with the Policy Circle (past or current members) and/or CFHI?

____

____

____

____

____

Q8 Was your work impacted by your participation in the Policy Circle? This includes if you have consulted or reffered to as a result of your involvement with the Policy Circle. (1 = Yes, significantly, 3= Yes, somewhat, 5=No, not at all)

- ◯ 1 (4)
- ◯ 2 (5)
- ◯ 3 (6)
- ◯ 4 (7)
- ◯ 5 (8)

Q9 Please explain your answer to the previous question.

____

____

____

____

____

Q10 Are you proud to tell others that "I am a Policy Circle member"? (1=Yes, 5=No)

- ○ 1 (6)
- ○ 2 (7)
- ○ 3 (9)
- ○ 4 (10)
- ○ 5 (11)

Q11 What can be done to support you as a Policy Circle Alumni?

____

____

____

____

____

Q12 Do you acknowledge your policy circle experience in current/or future work?

- ○ Yes (1)
- ○ No (2)

Q13 Please explain your answer to the previous question.

____

____

____

____

____

Q14 Lastly, please tell us your reflections on your experience as a Policy Circle member. If you can, provide an example or a short story that exemplifies your time as a Policy Circle member.

____

____

____

____

____

End of Block: Default Question Block

PC-C2-Midpoint

Start of Block: Default Question Block

Q7
Thank you for taking time to complete this survey. We are collecting this information as a quality improvement and self-evaluation exercise. You do not have to complete any questions you do not want to. There is no direct benefit to completing the survey, other than the chance to be reflective of your current engagement with the Policy Circle. Your individual results will not be shared. All data will be collected and reported as an aggregate. You will have a chance to review all the data with the policy circle in an upcoming meeting.
If you do have any questions, please reach out to Shannon (ssibbald@uwo.ca) or Bill (bill.callery@cfhi-fcass.ca).

Q2 In order, please list your top three goals for participating in the policy circle?

- ◯ 1. (7) ____
- ◯ 2. (8) ____
- ◯ 3. (9) ____

Q3
Which Policy Circle opportunities have you taken advantage of?

	Have you used this opportunity?		
	Yes (1)	No (2)	Not yet, but plan to (3)

Harkness readings (1)	○	○	○
Mentorship (2)	○	○	○
Quarterly meetings (3)	○	○	○
Attending conference (4)	○	○	○
IHI Open school (5)	○	○	○

Q4 Which other Policy Circle opportunities have you taken advantage of?

	Have you used this opportunity?		
	Yes (1)	No (2)	Not yet, but plan to (3)
Journal club (1)	○	○	○
CPSI/CFHI branding exercise (2)	○	○	○
Volunteer judging for CFHI's innovation challenges (3)	○	○	○
Other (4)	○	○	○

Q9 If you selected other - please explain:

____

Q7 What do you believe are the strengths of the Policy Circle program.

____

____

____

____

____

Q6 What can be done to improve the Policy Circle program?

____

____

____

____

____

End of Block: Default Question Block

PC-C2- Exit

Start of Block: Default Question Block

Q1
Thank you for taking the time to complete this survey. We are collecting this information as a quality improvement and self-evaluation exercise. You do not have to complete any questions you do not want to. There is no direct benefit to completing the survey, other than the chance to be reflective of your current engagement with the Policy Circle. Your individual results will not be shared. All data will be collected and reported as an aggregate. You will have a chance to review all the data with the policy circle in an upcoming meeting.
If you do have any questions, please reach out to Shannon (ssibbald@uwo.ca) or Bill (bill.callery@cfhi-fcass.ca).

Q2 Please list your top goal for participating in the Policy Circle?

____

____

____

____

____

Q3 In our first survey, we asked about your goals within the Policy Circle - here, we present an amalgamated list (in no particular order) of common goals. On a scale from 1-5 (1= Yes, significantly, 5 = No, not at all) did the Policy Circle program achieve the following goals?

	Did Policy Circle help you achieve the following?				
	1 (1)	2 (2)	3 (3)	4 (4)	5 (5)

Making new connections/ networking (1)	○	○	○	○	○
Mentorship (2)	○	○	○	○	○
Increased knowledge and skills / learning (3)	○	○	○	○	○
Accessing resources (4)	○	○	○	○	○
Professional Recognition (6)	○	○	○	○	○
Enjoyment of the program and the associated opportunities (7)	○	○	○	○	○
Sense of Belonging (10)	○	○	○	○	○

Q4 How many times (approx.) did you take advantage of the following Policy Circle opportunities?

	Number of times		
	0 (1)	1-2 (2)	3+ (3)

Harkness readings (1)	○	○	○
Mentorship Meetings (2)	○	○	○
Quarterly meetings (3)	○	○	○
IHI Open school (5)	○	○	○

Q5 Did you attend a conference?

☐ Yes (1)

☐ No (2)

Q6 If yes-which conference did you attend?

____

Q7 Did you take advantage of other Policy Circle opportunities?

	Yes or No	
	Yes (1)	No (2)

Journal club (1)	○	○
CPSI/CFHI branding exercise (2)	○	○
Volunteer judging for CFHI's innovation challenges (3)	○	○
Other (4)	○	○

Q8 If you selected other - please describe:

____

____

____

____

____

Q9 In our first survey we asked members what they believed were the strengths of The Policy Circle - Here we have compiled a list of the commonly mentioned strengths. Rank the following strengths in order from most to least important (1= most, 5 = least).

______ Networking (1)
______ Mentorship (4)
______ Program Coordinator for the Policy Circle (5)
______ Ability to advance knowledge (6)
______ Other (fill in) (7)

Q10 Explain how (if at all) your enthusiasm, engagement or expectations for the Policy Circle changed over the course of the past 12 months?

____

____

Q11 How would you like to continue your engagement with the Policy Circle?

Q12 Was your work impacted by your participation in the Policy Circle? This includes being consulted or referred to as a result of your involvement with the Policy Circle. (1 = Yes, significantly, 3= Yes, somewhat, 5=No, not at all)

- ○ 1 (1)
- ○ 2 (12)
- ○ 3 (13)
- ○ 4 (14)
- ○ 5 (15)
- ○ Unsure (17)

Q13 Please explain your answer to the previous question.

____

____

____

Q14 Are you proud to tell others "I am a Policy Circle member"? (1 = Yes, 4= No)

- 1 (6)
- 2 (7)
- 3 (9)
- 4 (10)
- Unsure (11)

Q15 In our first survey, we asked what could be done in the future to improve The Policy Circle program- Listed below (in no particular order) is a summary of the answers. Rank these Policy Circle improvement suggestions from most to least important (1 = most important, 8 = least important).
______ Adding face-to-face meetings in order to improve personal connection (1)
______ Extra monthly or quarterly meetings that focus on a specific topic (optional meetings) (2)
______ Inviting a guest speaker that members would not regularly get a chance to access (3)
______ Provide more information on how to access opportunities, like the IHI courses (4)
______ A collective goal from the start (6)
______ Have a formal contribution to policy that members can work on together throughout the year (7)
______ More structure, and linkages to different Canadian sector leaders in order to facilitate cross context engagement (8)
______ Other, please explain.. (9)

Q16 What can be done to support you as a Policy Circle member after the program is over?

____

Q17 How do you plan to acknowledge your Policy Circle experience in current and/or future work?

Q18 Lastly, please tell us your reflections of the past year as a Policy Circle member. Consider the impact of COVID. If you can, provide an example or a short story that exemplifies your time as a Policy Circle member.

End of Block: Default Question Block
